# Relevance of Vaccine Literacy Assessment Tools

**DOI:** 10.3389/ijph.2023.1605945

**Published:** 2023-03-24

**Authors:** Luigi Roberto Biasio, Patrizio Zanobini, Chiara Lorini, Guglielmo Bonaccorsi

**Affiliations:** ^1^ Scientific Advisory Committee, Giovanni Lorenzini Foundation, Milan, Italy; ^2^ Department of Health Sciences, School of Psychology, University of Florence, Firenze, Italy

**Keywords:** vaccine hesitancy, COVID-19, vaccine literacy, health literacy, vaccine uptake

The systematic review by Zhang et al. recently published on IJPH (1) provides an important update on the status of vaccine literacy (VL) and related measurement tools. While the Authors highlight the role of VL in overcoming vaccine hesitancy and increasing immunization rates, they also conclude that the tools used in the selected studies were “limited” in relation to the complexity of the influencing factors, as they were not designed to specifically assess VL skills, but were adapted from those used to measure health literacy (HL) in chronic patients (2).

Indeed, many and complex determinants influence VL, similar to HL where the role of individual, societal and environmental factors is still being explored, sometimes seen as moderating, sometimes as mediating (3). The same role of HL in mediating health outcomes has been under discussion. For VL this complexity seems even greater, given its relative novelty, and because it refers to a specific but broad science, “vaccinology,” addressing the domains of disease prevention and health promotion, and entailing the many topics related to vaccines, not only immunological and epidemiological, but also regulatory and social, including communication, economics, and ethics (4). Vaccinology has developed rapidly and massively in recent years, and has been defined differently, although an overarching definition is still being discussed. All these aspects show why the VL tools used to date may appear limited, especially when compared with more consolidated HL measures.

The same VL tools cited by Zhang et al. were used in the studies selected for our recent scoping review (5). We had also contributed to the development of the HLVa scale (Vaccine Health Literacy for adulthood) (6), including functional, interactive, and critical items, according to Nutbeam’s definition. HLVa was developed following earlier investigations where a modified version of the Ishikawa scale was used to assess parent’s VL levels about children vaccination (7, 8). Possibly, attitudes of participants in a literacy questionnaire may vary depending on whether it attempts to measure HL about the cure of an existing pathology, or about vaccination, more oriented towards prevention. However, we believe that there may not be much difference in whether individuals are considering an intervention as treatment or prevention, when they take part in a literacy survey. Therefore, in our opinion, it made sense to begin developing VL assessment tools based on consolidated self-rated measures for general HL.

In any case, the HLVa scale passed through face and construct validation, both performed before the COVID-19 outbreak, revealing two well-defined dimensions, i.e., functional and interactive-critical literacy. Afterwards, VL has received growing attention during the pandemic: literature has proposed other measures exploring VL skills about SARS-CoV-2, such as the COVID-19-VLS (-Vaccine Literacy Scale), adapted from HLVa, also including items assessing outcomes, such as attitudes, beliefs, and behaviors (i.e., vaccine acceptance) towards COVID-19 and other adulthood vaccines (9). The HLVa scale and COVID-19-VLS share the same psychometric construct, including functional, interactive (otherwise known as communicative), and critical questions. While HLVa includes 14 questions, in the case of COVID-19-VLS the items are reduced to a total of 12 (four for functional and eight for interactive-critical VL) to avoid redundancy, by eliminating two questions that resulted repetitive during the validation process of HLVa. Notably, the adapted scales are not short versions of the original HL tool. In adapting rating scales, a common practice to reduce survey time is to select some questions from an existing tool, even if it is known that maintaining a relatively high number in items reduces the risk of a possible impact on the construct validity and assessing potential of the original instrument (10).

**FIGURE 1:**
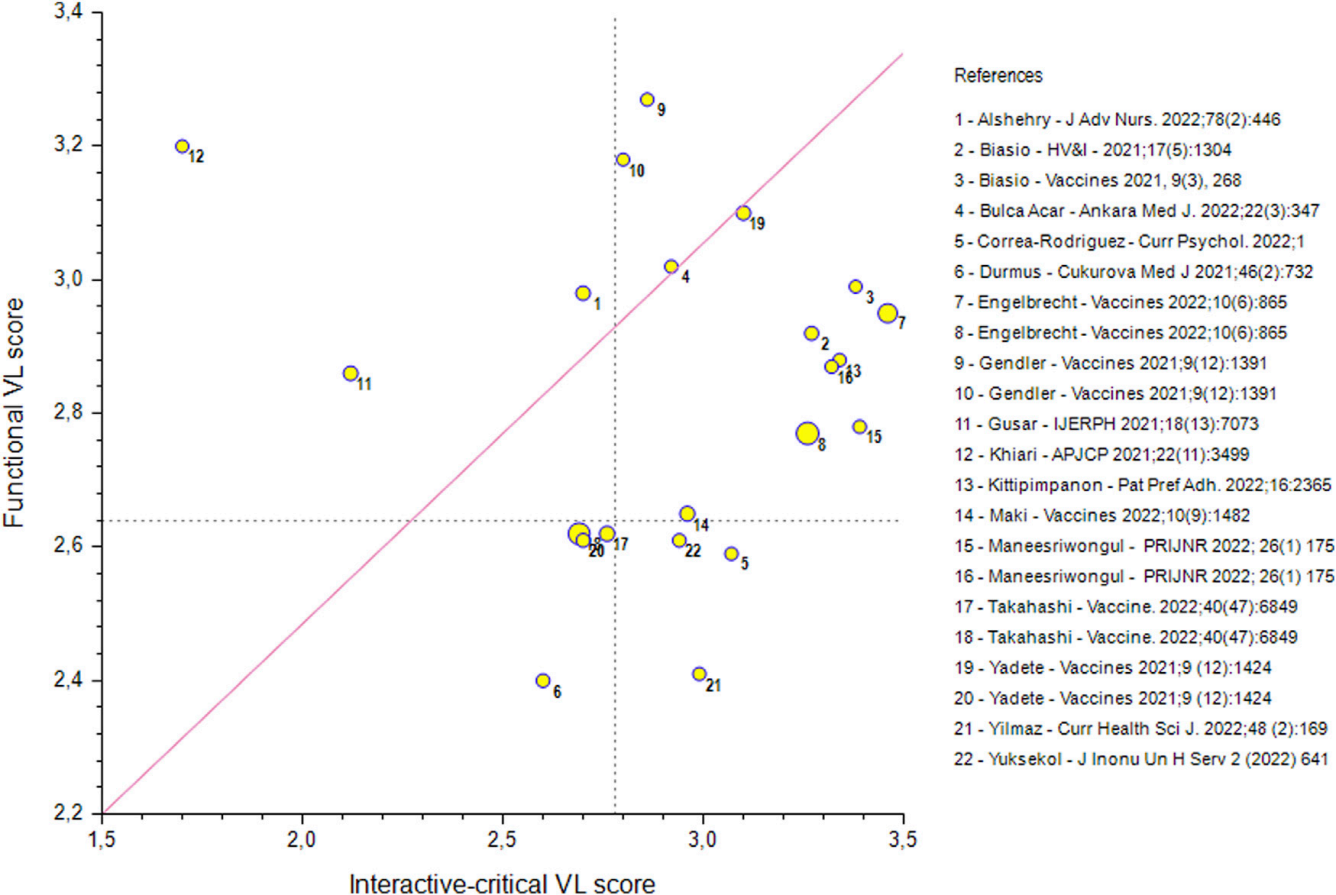
Scatter plot showing the functional and interactive-critical vaccine literacy (VL) scores observed in 22 adult general and patient populations from different countries, assessed by Covid-19-VLS or HLVa scales. The mean functional score was 2.83 (S.D. 0.25), whereas the interactive-critical one was 2.92 (S.D. 0.42), calculated on a four-point Likert scale, ranging from 1 to 4. The interactive-critical was greater than the functional VL values for 16 of the 22 populations. Marker dimensions reflects variability in the sample size of the studies (N between 154 and 6,275). Dotted lines refer to the proposed cut-off values for limited VL, corresponding to the lower tertile bounds. More details and full citations of the selected studies are reported in ref # (5). Scatter diagram of vaccine literacy scores (different countries, 2023).

While the scarcity of specific measures may have limited the assessment of VL skills, on the other hand the few available tools allowed performing comparisons. In fact, HLVa and COVID-19-VLS have been translated and validated into multiple languages, allowing to compare literacy skills in different populations, albeit only descriptively, given the heterogeneity of available data. Yet, this led to interesting observations, likely useful for future research as regards the actual construct of the tools. For example, the functional VL levels reported in the publications we selected were often lower than the interactive-critical VL levels, as if these were stimulated by the COVID-19-related “infodemic” (Figure 1). On the contrary, functional skills may have been challenged by complex terminologies and technical information provided by the scientific and lay media, which may explain the lower functional score, also among highly educated individuals. Of course, all these observations are partial and should be treated with caution since they come from various surveys and populations, even if comparable in terms of tools and scoring methods used. However, as reported in our review, factor loadings obtained by using the same extraction method (principal component analysis) and the same scale, i.e., Covid-19-VLS, showed no significant differences between populations of four different countries (Italy, Croatia, Thailand, and Japan), thus confirming the consistency of the two dimensions construct of the tool.

We agree with Zhang’s conclusions that all the specific dimensions of VL can possibly be underestimated, and that future research should be focused, among others, to develop more specific assessment methods to better determine the causal relationship between VL and vaccine hesitancy. However, we also believe that despite the possible limitations of the current VL tools, and even if the surveys selected for the reviews were mostly cross-sectional, and carried out primarily in the context of the pandemic, the accumulated experience remains important. Likely, coronavirus has changed the sentiment about the prevention of viral diseases in the general population, and produced lasting impact over time also with respect to the public perception of many other communicable diseases. The pandemic experience will in any case affect VL with regard to other vaccines, at least in the near future. Therefore, while the experience of VL tools used primarily during the COVID-19 outbreak may be considered limited, it provides a relevant starting point for future research.
